# The RISK pathway leading to mitochondria and cardioprotection: how everything started

**DOI:** 10.1007/s00395-023-00992-5

**Published:** 2023-05-26

**Authors:** Derek M. Yellon, Siavash Beikoghli Kalkhoran, Sean M. Davidson

**Affiliations:** The Hatter Cardiovascular Institute, University College London, 67 Chenies Mews, London, WC1E 6HX UK

**Keywords:** RISK pathway, PI3Kα, Mitochondria, MPTP, Ischaemia–reperfusion injury, Ischaemic preconditioning

## Abstract

Ischaemic heart disease, which often manifests clinically as myocardial infarction (MI), remains a major cause of mortality worldwide. Despite the development of effective pre-clinical cardioprotective therapies, clinical translation has been disappointing. Nevertheless, the ‘reperfusion injury salvage kinase’ (RISK) pathway appears to be a promising target for cardioprotection. This pathway is crucial for the induction of cardioprotection by numerous pharmacological and non-pharmacological interventions, such as ischaemic conditioning. An important component of the cardioprotective effects of the RISK pathway involves the prevention of mitochondrial permeability transition pore (MPTP) opening and subsequent cardiac cell death. Here, we will review the historical perspective of the RISK pathway and focus on its interaction with mitochondria in the setting of cardioprotection.

## Introduction

The incidence of myocardial infarction (MI) is in decline in developing countries. However, the morbidity and mortality resulting from MI remain high and may even start to increase as a consequence of the increasing prevalence of metabolic disease [18]. Hence, there remains an unmet need for the development of effective cardioprotective therapies to salvage the myocardium following an acute MI. Mitochondria are crucial organelles for the provision of ATP to maintain the viability and function of cardiomyocytes [40]. However, during myocardial ischaemia, mitochondrial respiration ceases. Subsequent reperfusion of the blocked coronary arteries contributes to mitochondrial-related oxidative insult, cell death, and inflammation [8, 82]. Hence, mitochondria have been extensively studied during the past few decades in the setting of ischaemia reperfusion injury (IRI) [22, 93]. In this context, cardioprotective signalling cascades are suggested to activate protein kinase C which subsequently regulates mitochondrial reactive oxygen species (ROS) and Ca^2+^ to prevent cell death following IRI [14, 15, 25, 41]. Relevantly, mitochondrial potassium ATP channels can mediate or initiate cell death by modulating mitochondrial ROS, and they are fundamental for the action of ischaemic preconditioning (IPC) [32, 65]. Therefore, mitochondria are crucial for the execution of various cardioprotective strategies.

In the early 2000s, Yellon and colleagues investigated the potential for growth factors such as transforming growth factor-beta1 (TGF-β1) and insulin to protect the heart from IRI via anti-apoptotic signalling pathways. In these initial studies, they demonstrated anti-apoptotic effects in both rat cardiomyocytes and isolated Langendorff perfused rat hearts when insulin or TGF-β1 administration was given at reoxygenation/reperfusion, following a lethal period of hypoxia/ischaemia, respectively. They further demonstrated that these agents mediated their effect via a number of pathways including the p70S6 kinase and phosphoinositide 3-kinase (PI3K) pathway as well as the ERK1/ERK2 mitogen-activated protein kinases (MAPK) signalling pathway, respectively. In these studies, they concluded that manipulation of growth factor “survival” signalling mechanisms may provide a promising route to attenuate lethal reperfusion injury [7, 42, 43].

Investigating this growth-factor signalling further using Urocortin, a growth factor which upregulates the ERK1/ERK2 (then known as p44 and p42 respectively, official names: MAPK3/MAPK1) MAPK signalling pathway, they demonstrated its ability to protect the heart against reperfusion-induced injury in isolated perfused and in vivo rat hearts [71]. Urocortin was given at the time of reperfusion following 35 min of ischaemia. The significant protection observed was associated with the upregulation of the ERK1/ERK2 MAPK-dependent signalling pathway [71]. As a result of these studies, they proposed that the heart possesses “prosurvival reperfusion injury salvage kinase” pathways that may be exploited when developing agents that can be used to protect the myocardium against the consequences of lethal reperfusion injury—the so-called RISK pathway. Importantly, the RISK pathway was defined as the kinases activated during early reperfusion [40]. The fact that intervention at or immediately prior to reperfusion was capable of limiting infarct size was important as it demonstrated that reperfusion injury exists.

Further studies used bradykinin as a cardioprotective agent to demonstrate that PI3K activity subsequently resulted in rapid phosphorylation of AKT/Protein kinase B (PKB) and endothelial NO synthase (eNOS) within the first 5 min of reperfusion, and that this was involved in attenuating reperfusion injury. This led to the proposal that salvaging the myocardium following IRI must involve the recruitment of the PI3K/AKT cell survival signalling cascade and an increase in eNOS activity to attenuate cell death during reperfusion [11].

Following these initial studies using growth factors, it was confirmed that activation of PI3K/AKT and ERK1/ERK2 MAPK pathways at reperfusion was essential for IPC [39]. Interestingly, ‘cross-talk’ was observed between these two pathways, whereby inhibiting one cascade activates the other and vice versa [38].

There is evidence that the extent to which the RISK pathway is recruited during cardioprotection varies between species. For example, the RISK pathway was shown to be required for the induction of cardioprotection by ischaemic post-conditioning (IPost) in rodents [46, 84] and small mammals [91], whereas it was dispensable in pigs [72, 77]. Interestingly, this lack of conservation between species does not appear to be unique to the RISK pathway, since PKC, whilst required for protection in small rodents and rabbits, appears to be dispensable for protection in pigs. [85]. Furthermore, the ability of IPC to activate the SAFE pathway to protect the hearts of pigs from IRI may be affected by genetic background [47]. Regardless of which kinases are required for ischaemic conditioning strategies in animals, the RISK pathway is required to protect human myocardium from simulated IRI by the use of either pharmacological cardioprotective agents or hypoxia/reoxygenation strategies [62, 75, 76].

Furthermore, cardiovascular comorbidities can directly or indirectly influence cardioprotection strategies, and in some instances, this has been shown to be via suppression of RISK pathway activation [9, 26]. For instance, chronic type 2 diabetes is known to abolish the cardioprotective effects of IPC by reducing AKT phosphorylation [70, 75, 83] and this is exacerbated by age [88]. Similarly, IPost has been shown to be ineffective in limiting IRI in spontaneously hypertensive rats [86].

As the RISK pathway kinases have several different isoforms and led to the phosphorylation and activation of a number of downstream kinases, it was important to ascertain whether any specific isoform was directly associated with transmitting the cardioprotective signal. There exist three different isoforms of PI3K, including PI3Kα, PI3Kβ, and PI3Kγ, which are expressed in cardiomyocytes [29]. In 2008, a study by Tsushima and his team showed that the genetic deletion of PI3Kγ can interfere with IPC signalling and exacerbate cardiac dysfunction following IRI. This was attributed to the inhibition of AKT and glycogen synthase kinase-3β (GSK-3β) phosphorylation in the mutant mice [5]. Similarly, later work by our group revealed that selective inhibition of PI3Kα by GDC–G326 can abrogate the cardioprotective effects of IPC confirming the essential role of PI3Kα in cardioprotective signal transduction [69]. In comparison to these two isoforms, the role of PI3Kβ in the RISK pathway is less understood. However, hearts carrying cardiomyocytes with specific deletion of the PI3Kβ isoform have been shown to have worse systolic function and exhibit higher levels of cell death following IRI versus their wild-type littermate [19]. Interestingly, endothelial deletion of PI3Kβ induced opposite effects in the hearts following IRI, which indicates the cell-specific function of this isoform in the heart [19].

The protein sequences of ERK1 and ERK2 are ~ 84% identical to each other in mammals and appear to have almost identical functions [17]. Early work by Fryer et al. showed that both ERK1 and ERK2 isoforms are activated by IPC within 5 min of reperfusion [27]. They also showed the phosphorylation of the ERK1 (P44) MAPK at reperfusion was greater than ERK2 (P42) MAPK [27]. However, a later study showed that infarct size increased in response to heterozygous deletion of ERK2 and homozygous deletion of ERK1 did not have any effects on infarct size [53].

AKT is activated in response to various physiological and stress stimuli, such as growth factors, hormones, hypoxia, and oxidative stress. We investigated which of the isoforms of AKT may be important downstream to PI3K in IRI [48]. This study showed for the first time that AKT1 but not AKT2 is an essential mediator of myocardial protection following IPC, i.e., only AKT1 plays the key role in transmitting the cardioprotective signal resulting in amelioration of myocardial injury as a result of severe IRI [48]. Collectively, these studies revealed the isoform-specific effects in the induction of cardioprotection mediated by different kinases of the RISK pathway.

## The RISK pathway and the MPTP

The role of mitochondria in cardiomyocyte death following IRI did not become evident until a series of experiments by Crompton and colleagues showed that isolated rat cardiac mitochondria can become lethally permeabilised due to the opening of a mitochondrial pore in the presence of Ca^2+^ and inorganic phosphate or in response to oxidative stress [20, 21]. Following these studies, in the early 1990s, Halestrap and colleagues documented that the mitochondrial permeability transition pore (MPTP) opens in the intact heart, in response to IRI [31]. They reported that, following the opening of non-specific pores, cardiac mitochondria undergo swelling and depolarisation. The pore opening was the result of reperfusion, and low pH during ischaemia limited its occurrence [31, 35]. Subsequent research showed that reperfusion causes calcium overload and oxidative stress, that together initiate MPTP opening [24]. These were the defining points that highlighted the importance of mitochondria in the pathogenesis of IRI and placed an emphasis on the detrimental role of reperfusion injury, rather than the ischaemia alone, following MI. Further research by Halestrap and others investigated the molecular composition of the MPTP and its mechanism of activation [3, 34]. They showed that the translocation of cyclophilin D (CYPD) from the mitochondrial matrix to the mitochondrial inner membrane was necessary for the induction of MPTP opening. This could be inhibited by cyclosporine A administered at the onset of reperfusion [34]. Most cardioprotective strategies that act via the MPTP also require the presence of CYPD. Although transgenic mice deficient in CYPD are protected against IRI, different studies have shown that IPC and IPost are unable to augment this protection due to the lack of the CYPD protein [37, 51]. To prove the potential clinical significance of the MPTP in the human heart, sanglifehrin-A (SfA), a related compound able to inhibit MPTP opening, was administered to surgically isolated human atrial trabeculae subjected to simulated IRI. Once given at the start of reoxygenation, SfA blocked the MPTP opening and enhanced functional recovery of the human atrial trabeculae following simulated IRI [73]. However, the direct translation of therapeutic benefits of MPTP inhibition in human patients has proven to be challenging, with two major clinical trials of MPT inhibitors conducted on patients with ST-segment elevation MI (CIRCUS and CYCLE) both returning neutral results [60, 68].

Although numerous studies shed light on the benefits of MPTP inhibition in the setting of cardioprotection, the link between cardioprotective signalling pathways such as RISK and the MPTP remained elusive. To demonstrate the downstream effects of the RISK pathway and its relation to the MPTP, Davidson et al. examined the hypothesis that the activation of the prosurvival kinase pathway could protect cardiomyocytes by reducing the probability of MPTP opening [23]. They showed that the overexpression of constitutively active AKT was sufficient to significantly delay MPTP opening, indicating that activation of the PI3K–AKT prosurvival kinase pathway inhibits the opening of the MPTP. These studies demonstrated an important link between the survival kinases and the MPTP [23].

The nature of the putative effector protein(s) that modulate the MPTP following the signal transduction via the RISK pathway remains a subject of debate. Juhaszova et al. were amongst the first groups to discover a common signalling route linking various cardioprotective treatments to the MPTP [44]. They showed that insulin can protect isolated cardiomyocytes subjected to hypoxia and reoxygenation (HR) by reducing MPTP opening, and this effect was abolished in the presence of an inhibitor of PI3K. The underlying mechanism behind this protection was attributed to the increased phosphorylation of GSK-3β at Serine 9 (Ser9), thereby inhibiting its activity. Insulin’s effects were similar to the protection achieved in cardiomyocytes by eliminating GSK-3β (but not GSK-3α), thereby emphasising the importance of GSK-3β inactivation in the induction of cardioprotection by the RISK pathway [44]. In contrast to this report, Marber et al. verified the role of GSK-3β in the same setting and developed transgenic GSK-3β mice lacking both the critical Ser9 and Ser21 phosphorylation sites [63]. They showed that GSK-3β knock-in heart could be still protected against MPTP opening by insulin, IPC, and IPost treatments, thereby raising questions about the importance of GSK-3β function in the induction of the MPTP. Instead, they proposed that these cardioprotective treatments may block the MPTP by reducing oxidative stress at the onset of reperfusion [63]. A more recent study utilised both genetic and pharmacological approaches to document that GSK-3β inhibition additionally incorporates mechanisms independent of the MPTP such as autophagy to elicit cardioprotection [96]. Therefore, the cardioprotective effects of GSK-3β inhibition may go beyond the blockade of the MPTP. Several other candidate proteins such as those that participate in mitochondrial dynamics have been suggested to bridge the gap between the RISK pathway and MPTP induction. These proteins have been shown to be post translationally modified by the kinases of the RISK pathway which allows them to confer protection by reducing ROS, MPTP opening, and infarct size following IRI [45, 56, 66, 67]. Moreover, accumulating evidence during the past decade has documented the role of F_O_F_1_-ATPase in modulating MPTP opening during ischaemia and reperfusion [1, 13, 61]. However, the interplay between the RISK pathway and subunits of F_O_F_1_-ATPase remains elusive.

## PI3K activation and cardioprotection

**Fig. 1:**
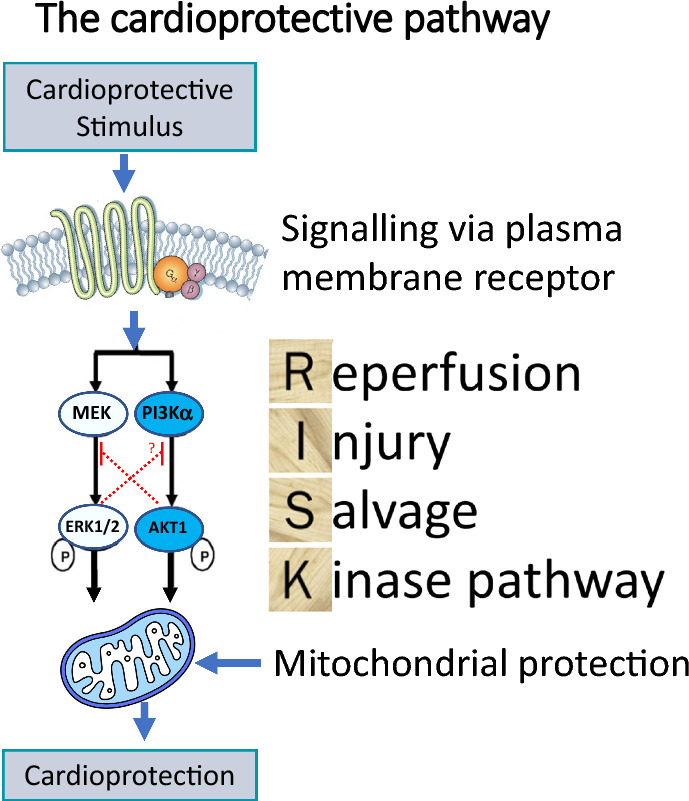
The cardioprotective pathway

PI3K is central to the induction of the RISK pathway and major cardioprotective signalling molecules, as summarised in Table 1, utilise its function to induce their effects (Fig. 1). Since little was known about the roles of the individual isoforms of PI3K in cardioprotection, studies were designed to elucidate which isoform played a significant role in the protection observed following IRI. The main focus was placed on PI3Kα, since it was shown to play a central role in cardioprotection by participating in cardiac physiology, improving contractility, and promoting physiological exercise-induced growth but not pathological hypertrophy [59]. A study by Tsushima and his team showed that mice with PI3Kα dominant negative hearts, lacking 77% of PI3Kα activity, were resistant to IRI and this protection was lost in PI3Kγ^−/−^ mice [5]. The protective effects in PI3Kα-dominant negative hearts were therefore attributed to the compensatory upregulation of PI3Kγ. However, the same group showed that heterozygous PI3Kγ^+/−^ mice were also resistant to myocardial IRI. In addition, it was not shown whether complete deletion of PI3Kα was beneficial to the heart [5]. Conversely, double-mutant mice carrying PI3Kγ^−/−^ and cardiac-specific PI3KαDN were later shown to have enhanced recovery following the IRI [57]. Contrary to these findings, constitutively active PI3Kα was demonstrated to improve left-ventricular function in heart failure, and indeed, the PI3Kα activator, insulin, was proven to be cardioprotective via PI3K [58, 92].

**Table 1:** A selected list of some of the major signalling molecules, peptides, and drugs that have been shown to induce cardioprotection by activating PI3K in the setting of myocardial IRI

Category	Molecule
Adipokines	Leptin [78]
	Visfatin [52]
	Apelin [74]
	Adiponectin [33]
	Resistin [28]
Anti-diabetic and anti-hypercholesterolaemic drugs	Atorvastatin [10]
	Pioglitazone (a thiazolidinedione and PPARgamma receptor agonist) [89]
	Metformin [12]
Autocoids	Bradykinin [11]
	Adenosine [36]
Chemokines	Stromal cell-derived factor (SDF) [16]
Extracellular vesicles	Exosomes [2, 81]
Growth factors	Cerebral dopamine neurotrophic factor (CDNF) [55]
	Insulin-like growth factor-1 (IGF-1) [50]
	Neuregulin-1 [87]
	Fibroblast growth factor 21 (FGF-21) [54]
	Granulocyte colony-stimulating factor (GCSF) [80]
	Hepatocyte growth factor (HGF) [90]
	Growth factor releasing hormone (GFRH) [30]
Hormones	Erythropoietin [62]
	Triiodothyronine [95]
	Insulin [4]
	Melatonin [94]
	Glucagon-like peptide-1 (GLP-1) [6]
	17beta-estradiol (E(2)) [79]
	Adrenomedullin [64]

To address these controversial findings and based on the notion that insulin has been shown to be a canonical activator of PI3Kα, we took a pharmacological approach to investigate the role of PI3Kα isoform in cardioprotection. Using insulin to activate PI3Kα, in combination with highly selective inhibitors of PI3Kα, Rossello et al. undertook a series of experiments using the isolated perfused mouse heart subjected to IRI as well as immortalised mouse cardiac endothelial cells, cardiomyocytes, and human tissue to ascertain the role of pharmacological activation of PI3Kα activation in cells and tissues [69]. In summary, these studies demonstrated that activity of the PI3Kα isoform activity was required, during the early reperfusion phase, to reduce myocardial infarct size. This led to the conclusion that the development of drugs, specifically enhancing PI3Kα activity at reperfusion, could potentially promote myocardial salvage in patients undergoing acute MI [69].

If the above is correct, then harnessing the potential beneficial effects of kinase signalling through the generation of an isoform-specific PI3Kα activator could be of direct benefit to patients presenting with an acute MI. To develop such highly specific PI3Kα activators, we undertook a series of experiments with a number of collaborators at UCL (Gong et al. Nature 2023 in press, 10.1038/s41586-023-05972-2) in which we conducted an unbiased high throughput screen on 450,000 compounds from the AstraZeneca screening library with the aim of identifying small molecules that could activate the in vitro lipid kinase activity of recombinant human p110α/p85α, namely PI3Kα. Medicinal chemistry (at UCL) was then used to increase in vitro potency (as measured by in vitro activity on recombinant PI3Kα) and cellular potency (as measured by AKT phosphorylation in human A549 cells), all of which led to the generation of UCL-TRO-1938—referred to as 1938. We demonstrated that 1938 was able to induce the PI3Kα pathway in mouse primary cells as well as demonstrate infarct size limitation using both isolated ex vivo and in vivo rat heart models subjected to IRI. Further studies are now underway to assess the exact impact of UCL-TRO-1938 on the signalling cascade of the RISK pathway and MPTP.

## Conclusions and perspective

The results discussed above represent the culmination of more than 3 decades of research in the field of cardioprotection that have highlighted the significance of the RISK pathway in the treatment of IRI. There is now extensive pre-clinical evidence that the activation of the RISK pathway, by a range of pharmacologic agents or by mechanical interventions such as IPC or IPost, reduces MI size by up to 50% via modulation of the MPTP. In accordance with the step-by-step criteria for IMproving Preclinical Assessment of Cardioprotective Therapies (‘IMPACT’) [49], the next steps are now to investigate the efficacy of RISK pathway activation in small animal models in the presence of potentially confounding comorbidities, such as age or diabetes [26], and the large animals of IRI.

## Data Availability

No data was generated for this article.
